# Development of an antimicrobial stewardship implementation model involving collaboration between general practitioners and pharmacists: GPPAS study in Australian primary care

**DOI:** 10.1017/S1463423620000687

**Published:** 2021-01-28

**Authors:** Sajal K. Saha, David C.M. Kong, Karin Thursky, Danielle Mazza

**Affiliations:** 1 Department of General Practice, Monash University, Notting Hill, Victoria, Australia; 2 National Centre for Antimicrobial Stewardship (NCAS), The Peter Doherty Institute for Infection and Immunity, Melbourne, Victoria, Australia; 3 Centre for Medicine Use and Safety, Monash University, Notting Hill, Victoria, Australia; 4 Department of Medicine, University of Melbourne, Melbourne, Victoria, Australia; 5 Pharmacy Department, Ballarat Health Services, Ballarat, Victoria, Australia

**Keywords:** Antimicrobial stewardship, community pharmacists, general practitioners, interprofessional collaboration

## Abstract

**Background:**

Rising antimicrobial resistance (AMR) in primary care is a growing concern and a threat to community health. The rise of AMR can be slowed down if general practitioners (GPs) and community pharmacists (CPs) could work as a team to implement antimicrobial stewardship (AMS) programs for optimal use of antimicrobial(s). However, the evidence supporting a GP pharmacist collaborative AMS implementation model (GPPAS) in primary care remains limited.

**Aim:**

With an aim to design a GPPAS model in Australia, this paper outlines how this model will be developed.

**Methods:**

This exploratory study undertakes a systematic review, a scoping review, nationwide surveys, and qualitative interviews to design the model. Medical Research Council (MRC) framework and Normalization Process Theory are utilized as guides. Reviews will identify the list of effective GPPAS interventions. Two AMS surveys and paired interviews of GPs and CPs across Australia will explore their convergent and divergent views about the GPPAS interventions, attitudes towards collaboration in AMS and the perceived challenges of implementing GPPAS interventions. Systems Engineering Initiative for Patient Safety (SEIPS 2.0) model and factor analyses will guide the structure of GPPAS model through identifying the determinants of GPPAS uptake. The implementable GPPAS strategies will be selected based on empirical feasibility assessment by AMS stakeholders using the APEASE (Affordability, Practicability, Effectiveness and cost-effectiveness, Acceptability, Side-effects and safety, Equity) criteria.

**Discussion:**

The GPPAS model might have potential implications to inform how to better involve GPs and CPs in AMS, and, to improve collaborative services to optimize antimicrobial use and reduce AMR in primary care.

## Background

Rising antimicrobial resistance (AMR) is an increasing concern in primary care due to overprescribing and inappropriate use of antimicrobials in this setting (Costelloe *et al.*, 2010). Therefore, promoting antimicrobial stewardship (AMS) programs in primary care is essential to optimize antimicrobial use and prevent AMR.

AMS is defined as the coordinated interventions or programs that aim to achieve optimal patient outcomes through limiting adverse events and promoting the selection of antimicrobial(s) with optimal choice, dose, duration and route of administration to prevent AMR (Infectious Disease Society of America, 2019). Evidence shows that the implementation of AMS programs depends on the availability of AMS related guidelines, training, resources and more importantly the system structures that facilitate interprofessional engagement and collaboration (Gebretekle *et al.*, 2018). These are significantly lacking in primary care including a lack of doctor–pharmacist collaboration that potentially hinders AMS implementation. Though doctor–pharmacist collaborative models for AMS exist in hospital settings, it is unrealistic to utilize these in primary care due to divergence in the routine practice environment, resources, organizational challenges, and interprofessional team functioning between two health care settings.

General practitioners (GPs) and community pharmacists (CPs) are the most important antimicrobial stewards in primary care (Bishop *et al.*, 2019). They are the first point of contact to the patients with infections and CPs are well positioned to provide comprehensive antimicrobial care through liaison with GPs. In this regard, an effective collaboration between GPs and CPs is the key for CPs to firmly engage in AMS activities. To date, a collaborative care model involving GPs and CPs to promote AMS is potentially limited globally including in Australia (Zingg *et al.*, 2019).

The Australian Commission on Safety and Quality in Health Care has recommended to establish AMS programs in primary care under the national AMR strategy 2015–2019 (ACSQHC, 2019). As part of the initiative, the Australian government has funded the National Prescribing Service (NPS) Medicine Wise to conduct a list of AMS related educational programs targeting GPs and CPs. Examples include ‘antimicrobial awareness week’ and ‘resistance fighter campaign’, online modules related to antimicrobial prescribing, continuous professional development (CPD) activities, and media releases on AMR and antimicrobial use (ACSQHC, 2018; National Prescribing Service Medicine Wise, 2018). However, whether and how GPs and CPs have been responding to these nationally promoted strategies remain unclear.

According to a 2019 report (ACSQHC, 2019), two in five Australians were dispensed at least one antimicrobial, 50% of antibiotic prescriptions were ordered with repeats and dispensed within 10 days. Despite no evidence of benefits, antibiotics were prescribed in 92% of acute bronchitis patients. The guideline adherence of the prescribed antibiotics varied from 27% for sinusitis to 67% for pneumonia (ACSQHC, 2017). These data demonstrate that how important it is to develop a sustainable AMS implementation strategy in Australian primary care involving GPs and CPs.

A systematic review and meta analysis (Saha *et al.*, 2019b) reported that AMS strategies involving pharmacists can reduce antibiotic prescribing and improve guideline–adherence of prescribing by GPs. Effective interventions included group meetings between GPs and pharmacist, pharmacist facilitated academic detailing, educational training, and audit and feedback. Besides, a collaborative program between GPs and CPs to manage influenza (Klepser *et al.*, 2016b) and pharyngitis (Klepser *et al.*, 2016a) showed significant benefits in reducing the use of antibiotics. These programs utilized the point of care tests using a collaborative practice agreement between general practice and pharmacy (Cooke *et al.*, 2015; Klepser *et al.*, 2016a, 2016b). The participation of CPs in those collaborative AMS programs is limited due to limited collaborative practice agreements between the GP practice and community pharmacy (Saha *et al.*, 2019a).

This study aims to develop an AMS implementation model involving GPs and CPs (GPPAS) in Australian primary care addressing the following research questions which have been built upon our systematic review (Saha *et al.*, 2019b) demonstrating evidence of effective AMS strategies that could be implemented by collaboration between GPs and CPs. This paper outlines how the GPPAS model will be developed.

## Research questions

1. What are the views of GPs regarding the awareness, use of evidence-based AMS strategies, collaboration with pharmacists in AMS, and future improvement strategies to improve AMS?
2. What are the views of CPs regarding the awareness, use of evidence-based AMS strategies, collaboration with GPs in AMS, and future improvement strategies to improve AMS?
3. What do GPs and CPs perceive as the barriers and facilitators to implementing AMS in practice?
4. What is the convergent and divergent views of GPs and CPs regarding AMS programs and their collaborative AMS implementation approaches?
5. What are the views of stakeholders about the implementation feasibility of evidence-based GPPAS intervention strategies in Australian primary care?
6. How to design a GPPAS model to optimise antimicrobial use in Australian primary care?

### Theoretical framework

The Medical Research Council (MRC) framework (Craig *et al.*, 2008) and the Normalization Process Theory (NPT) (Murray *et al.*, 2010) will be used as guides for developing the GPPAS model. Systems Engineering Initiative for Patient Safety (SEIPS 2.0) model will help identify the GPPAS model components including the determinants that would influence the implementation of GPPAS model.

### MRC framework

To develop and evaluate a complex intervention, a strong theoretical foundation is essential to describe interventions and explore societal context that influences intervention success (Hardeman *et al.*, 2005; Michie *et al.*, 2009). This framework identifies the complexity of designing intervention. The complexity involves in the health care practices at the process level and in the level of interactions among health care providers and patients (Craig *et al.*, 2008; Moore *et al.*, 2015). The MRC framework consists of four different phases that include development phase, feasibility and piloting phase, evaluation phase, and the implementation phase (Craig *et al.*, 2008). We use this framework as a guide for the development phase of the GPPAS model in particular to define and understand the AMS implementation problem in primary care and to identify evidence-based AMS strategies that could be implemented by fostered collaboration between GPs and CPs.

### NPT framework

The NPT framework uses ranges of theoretical approaches and methods appropriate to the policy questions and considers the wider societal context in which interventions are to be deployed (Murray *et al.*, 2010). It also identifies the factors that promote and inhibit the routine incorporation of complex interventions into everyday practice. Additionally, it helps to explain how complex interventions work to the point where intervention becomes so embedded that it disappears from views (normalized). Furthermore, it focuses on the work that enables intervention recipients to make intervention(s) become normalized. NPT grounds four theoretical constructs that explain implementation mechanisms: coherence (sense making of interventions), cognitive participation (engagement with intervention), collective actions (work done to enable intervention to happen), and reflexive monitoring (cost benefit appraisal). The major significance of using this framework is that its four components maintain a dynamic relationship with the social and organizational context, structural norms, group process, and conventions. This framework will guide how to critically assess whether and how GPs and CPs would be able to implement the complex GPPAS interventions in practice.

## Methods

This study has been framed with four major components: literature reviews (a systematic review and a scoping review), nationwide surveys, qualitative interviews, and an empirical feasibility assessment study.

### Component 1: literature reviews

#### Systematic review

We developed a systematic review protocol (Saha *et al.*, 2018), conducted the review (Saha *et al.*, 2019b), and determined what and whether interventions involving pharmacists are effective to reduce antibiotic prescribing rate (APR) and improving guideline adherent antibiotic prescribing rate (APAR) by GPs. The effective interventions with their effect sizes are detailed in the published review (Saha *et al.*, 2019b). In briefly, we identified 35 studies but 15 studies were eligible for meta-analysis. Only one study was obtained from Australia. We found APR reductions (Odds Ratio 0.86 and 95% CI 0.78–0.95), and APAR improvements (Odds Ratio 1.96 and 95% CI 1.56–2.45) when interventions were implemented by a GP–pharmacist team. Effective interventions identified were: GP education plus prescribing feedback, GP–pharmacist group meetings, team-based academic detailing, guideline development and use, audit and feedback, and workshop training. However, their contextual usability, acceptability, and feasibility are still unclear, an area that needs further exploration to understand how best GPs and CPs can be engaged to implement those effective strategies for optimal use of antimicrobial(s) in primary care.

#### Scoping review

We carried out a systematic scoping review (Saha *et al.*, 2019a) at global perspective to: identify the extent of AMS survey studies conducted at community pharmacy context, reveal what AMS strategies are used by CPs, and understand the perceived barriers and facilitators to implement AMS by CPs. The review findings details are found in Saha *et al.* 2019a.

The known evidence-based AMS strategies informed by above reviews, where there is a scope of GPs and CPs to work together, will be explored in the following surveys to assess their uptake and implementation challenges in the context of Australian general practice and community pharmacy.

### Component 2: nationwide surveys

#### Methods

We will conduct two nationwide surveys separately for GPs and CPs across Australia. A survey reporting guideline described by Pulcini and Leibovici, 2016 will be used to report the surveys.

#### Development of survey instruments

Survey instruments were designed in collaboration with experienced AMS researchers from the National Centre for Antimicrobial Stewardship (NCAS) and the Department of General Practice of Monash University, Australia. Six AMS surveys targeting GPs (Baadani *et al.*, 2015; Giry *et al.*, 2016; Mauffrey *et al.*, 2016; Rodrigues *et al.*, 2016; Owens *et al.*, 2017; Zhuo *et al.*, 2017) and nine CP-AMS surveys (McNulty, 2012; Pawluk *et al.*, 2015; Erku, 2016; Hancock and Mellor, 2016; Khan *et al.*, 2016; Avent *et al.*, 2018; Jamshed *et al.*, 2018; Rizvi *et al.*, 2018; Sarwar *et al.*, 2018) and reviews of study component 1 were used to develop survey questionnaires. The questionnaires were reviewed by AMS expert team of GPs (DM), infectious disease specialists (KT), pharmacists (DK), and AMS researchers for the clarity, relevance and importance of the contents. After adjustments were made to the questionnaire, readability was further checked by AMS and non AMS researchers.

#### Description of survey questionnaire

The final survey instruments consist of 34 quantitative items (GP survey) (Supplementary File 1) and 40 quantitative items (CP survey) (Supplementary File 2). Both instruments had two open-ended questions related to barriers and enablers to implement AMS. The survey instruments had 20 identical items for both GPs and CPs to determine the commonalities and differences in the views of GPs and CPs regarding awareness, practices, collaboration, and improvement strategies concerning AMS. Featured structures of survey instrument are described in Table 1.

**Table 1.** Featured structures of survey instruments

**1. Demographics**
Demographic information covering gender, level of education, practice experiences, practice location, working territories, medical/pharmacy training inside or outside of Australia and whether the participants completed the antimicrobial modules developed by the National Prescribing Services (NPS) Medicine Wise in Australia.
**2. Items relating to AMS awareness**
AMS awareness refers to the familiarity of GPs and CPs with the term AMS, its importance on reducing inappropriate use of antibiotics and costs associated with infections, the impact of the GPs’ and CPs’ individual efforts on AMS, and training needs required to undertake AMS.
**3. Items highlighting AMS activities currently undertaken in practices**
These items focused on the known AMS strategies like the use of guidelines, point of care tests, and patient leaflets. Besides, the adoption of strategies like educating patients, recording clinical indication, discussing antibiotic prescribing reports, and auditing antimicrobial prescriptions and providing feedback to improve the quality of prescribing, reviewing GPs antimicrobial prescription by CPs followed by contacting GPs if necessary.
**4. Items exploring attitude towards collaborations between GPs and CPs in AMS**
The collaboration items will highlight the topic of relevant policy changes, GPs’ receptiveness towards CP’s recommendations on the choice, dose, and dose–regimen of antibiotic prescriptions, allowing pharmacists to conduct regular meetings with GPs, pharmacist’s co-location, the impact of using ‘My Health Records’, reviewing antimicrobial prescriptions, and utility of prescription exchange technology to drive collaboration.
**5. Items canvassing the attitude towards proposed AMS intervention measures and policies to better improve antimicrobial use in primary care**
This construct focused GPs’ and CPs’ attitudes on the evidence-based AMS strategies, intervention needs and policy supports in the future to improve the uptake of AMS strategies during patient care.
**6. Open-ended questions exploring the beliefs of GPs and CPs regarding the major barriers and facilitators to implementing AMS.**
The aims of including these open-ended questions were to give GPs and CPs the freedom to express exactly how do they feel about the practical AMS implementation barriers and gather unique ideas at personal or organisational or policy level that may reveal unforeseen opportunities and issues with regards to AMS implementation either in general practices or community pharmacies or through GP pharmacy collaboration. This information is assumed to increase the credibility and utility of quantitative findings.

#### Pretesting

The survey instruments were pretested by practising GPs (*n* = 7) and CPs (*n* = 7) to ensure internal consistency, content validity, and detecting any sources of errors. Participant GPs and CPs provided feedback on comprehensibility and construction, design, structures of questionnaires, relevance, and duration. Feedback returned was incorporated into the final design of the survey tools.

#### Sampling, recruitment and survey deployment

There are 34,606 GPs (Department of Health, 2017) and 12,000 CPs in 5700 pharmacies (Pharmacy Board of Australia, 2018) in Australia. A total sample size of 381 GPs and 373 CPs will be required to afford 80% power, 50% response distribution (to get the largest sample size required), 95% confidence level, and 5% margin of error (Raosoft, 2018).

The AmpCo database, a commercially available database of Australian health professionals (Gattellari *et al.*, 2008) will be the source of GPs’ contact addresses. This database ensures the national representativeness of health professionals in terms of socio demographics and gender. The source of contact addresses of 2160 community pharmacies will be the public websites of pharmacy authorities across six states and two territories in Australia.

A multistage sampling method will be used to select GPs and pharmacies (Supplementary File 3). In the first stage, stratification of practising GPs will be employed based on GPs’ location of work in Australia [six states (NSW, QLD, SA, TAS, VIC, and WA) and two territories (ACT and NT)]. In the second stage, 3000 GPs will be randomly selected using probability proportionate to GPs’ size in each territory (Supplementary File 3). For recruiting pharmacies, we will first stratify community pharmacies based on the location of pharmacies across six Australian states and two territories. Then, we will randomly select a total of 2160 community pharmacies across Australia using probability proportionate sample size in each state and territory using a simple random sampling technique.

Upon receiving ethics approval, 3000 GPs and 2160 community pharmacies will be invited to participate via mail comprising of a package of an invitation letter, an explanatory statement, a survey questionnaire, and a reply paid envelope. Non-responders will receive two reminders at approximately three weeks intervals. GPs’ responses will be treated as implied consent. CPs will be requested to participate in either the postal survey or through an online survey link. The REDCap, web-based application software of Monash University was used for building and managing the online version of CP survey. CPs’ responses will be treated as implied consent. All respondents of GPs and CPs will be eligible to win one of four gift vouchers ($100).

## Data analysis

Only respondents who complete the demographic questions and at least one other construct will be included in the final data analyses. Data will be cleaned by double manual data entry methods (Paulsen *et al.*, 2012). Questions with 5 point Likert (‘strongly disagree’ to ‘strongly agree’ and ‘always to never’) type responses will be measured on a 5 point scale from 1 to 5. The cumulative scores for the attitudinal constructs will be calculated. Given the sample size is smaller than 10 for any construct, non parametric tests such as Fisher’s exact test and Kruskal–Wallis test will be performed.

Descriptive statistics will be used to examine the frequencies and percentages of responses for both demographics and survey constructs. The response rate will be calculated for all demographic variables. Response rates for the variables such as Australian states, remote rural locations, and gender will be compared with the national GP workforce (Department of Health, 2017) and the pharmacy workforce (Pharmacy Board of Australia, 2018) statistics to observe the degree of representativeness of the data. Medians, interquartile ranges (IQRs) and the rank sum will be quantified and reported for each of the constructs on an ordinal response scale.

The normality of the data will be assessed by the Shapiro–Wilk test. Given the data supports normality, linear regression, and multivariable linear regression model will be employed to determine the significant predictors of awareness of AMS, AMS practices during prescribing and dispensing of antimicrobials, and collaboration between GPs and CPs in AMS. If data do not comply with the normality, independent sample Kruskal–Wallis tests, and Mann–Whitney U tests, and multiple logistic regression will be employed to determine the factors influencing awareness of AMS, AMS practices, and GP pharmacist collaboration. The results of logistic regression analyses will be presented by odds ratio (OR), 95% confidence interval (CI) and p-value. Comparing proportion Chi square tests will be used to compare the commonalities and differences in views of GPs and CPs with regards to AMS awareness, practices, and collaboration using the responses of 20 identical items of both survey tools. A minimum total sample size of 336 (112 in one group and 224 in another group) will be required to detect a difference between two proportions of at least 15 for an α level, 0.05 and β level, 0.20 with affording 80% power.

A Cronbach’s alpha reliability coefficient (Santos, 1999) will be calculated for each construct and the complete survey tools. Principle component analysis (PCA) (Jolliffe, 2011) will be performed to find factors influencing the adoption of AMS strategies and attitudes of interprofessional collaboration in implementing AMS. Factors from PCA will be extracted at eigenvalue >1. The PCA results of GP survey and CP survey will guide the development of the structural model for interprofessional collaboration between GPs and CPs in AMS. Structural equation modeling (Holmes-Smith *et al.*, 2006) will be used to determine how attitudes of participant GPs and CPs influence collaboration behaviors in implementing AMS. The adequate model fit of our proposed model will be assessed using Relative Chi Square (χ2/df) (χ2/df <3), Root Mean Square Error of Approximation (RMSEA) with 90% confidence level (RMSEA < 0.08), and Bentler Comparative Fit Index (CFI) (CFI >0.90). Microsoft Excel and IBM SPSS Statistics V.24 will be used to perform all statistical analyses.

Free text data regarding the barriers and facilitators to implementing AMS will be analyzed using a framework of human factor engineering model, SEIPS 2.0 (Holden *et al.*, 2013; Keller *et al.*, 2018). Two coders will code the data independently. Data will be coded using excel and NVivo 11 based on the data.

### Component 3: paired interviews

This study would explore the views of GPs and CPs regarding the selective evidence-based collaborative AMS strategies to deeply understand the aspects of implementation challenges and needs to normalize those interventions in routine practices. A list of selective AMS strategies will be identified using the results of reviews and nationwide surveys. The factors that might affect the collaborative implementation of those AMS strategies either attitudinal or organizational or policy level will be explored. Consideration of these factors would be important to target interventions, and design and refine the planned GPPAS model.

## Study design

This qualitative study will be undertaken using an online paired in depth interview method as described by Wilson *et al.*, 2016. A researcher along with an assistant will interview each GP pharmacist pair online using Zoom teleconferencing. AMS strategies that have been proved as effective in another context will be discussed in paired interviews to collect information about their usability, feasibility, implementation barriers, and probable collaborative models for implementation in practice. GPs working in general practice and pharmacists working in community pharmacy will only be included. Pharmacists working in hospital settings will be excluded.

## Sampling and recruitment

We anticipate running 20 paired interviews recruiting 40 participants (20 GPs and 20 pharmacists). Each paired interview will include one GP and one pharmacist. Among the participants who will respond to AMS surveys and agree to participate in paired interviews will be identified. We will further confirm their consent for participation using mail or email where possible and will select for paired interviews. To arrange a common interview time for the dyad (a GP and a pharmacist) groups, both GPs and pharmacists will be asked to fill their preferred date and time in a structured doodle poll that will present options from Monday to Friday and ranks availability. Those GPs and pharmacists who match their time and date will be paired. Agreed participants will be confirmed and will receive reminders before one week and 24 h before the interviews. If any participant drops out, a new pair will be formed if possible and also new date will be fixed for interviews. Total pairs will be dependent on data saturation principles.

## Interview schedules and strategies

The NPT will be used to develop the interview guides. Four component NPT model will be employed to understand the implementation of AMS interventions and wider issues of implementation like the social and organizational context, structural norms, group process, and conventions in which interventions are to be deployed. Four components of NPT include coherence (sense making), cognitive participation (engagement), collective action (operationalisation), and reflexive monitoring (appraisal). Thus, the interview guide will be designed to explore four major areas: how do GPs and pharmacists make sense (degree of understanding over the purpose and needs) of selective AMS strategies and what is their feedback?; how implementable are these strategies by their engagement (investment of efforts) at primary care context?; what do participants believe are the operational barriers and facilitators of implementing each of the selected AMS strategies?; and how do participants appraise (evaluations) collaborative models for the implementation of AMS strategies in practice? To promote discussion, AMS-related documents or video may be incorporated. At the end of the interview, participants will be requested to rank selective AMS strategies based on their thoughts on the implementation feasibility from the context of their existing practices.

## Data collection

All interviews will be conducted by a research assistant and an assistant note taker (NT). Four types of qualitative data (Wilson *et al.*, 2016) will be collected. They include individual verbal data from GPs and pharmacists, GP–pharmacist dyad verbal data, GP–pharmacist dyad interaction data (reporting by NT), and nonverbal communication data for GPs and pharmacists (reporting by NT). The purpose of this method of data collection will be to observe the nature and dynamics of the GP pharmacist relationship, how GPs and pharmacists compromise to make collaborative decisions, triangulate their experiences, and to determine their future willingness for collaboration in relation to implementing the proposed AMS strategies in practice. The dyad nature of the interviews may produce some meaningful themes to support a collaborative AMS care model. Each interview will last up to 1 h. Interviews will be audio and video recorded and transcribed verbatim.

## Data analysis

A framework analysis approach (Ritchie and Spencer, 1994) and an analytical technique of qualitative comparative analysis (Wilson *et al.*, 2016) will be used to analyse all four types of data. This method will systematically analyse the differences and similarities in views between dyad members of GPs and pharmacists to build a theory of collaborative care models. Emerging themes from the verbal data will be explained by the NPT to develop the implementable GP–pharmacist collaborative AMS intervention strategies in the context of the Australian primary care. Data will be imported into NVivo11 software for analyses, and be deidentified. Approximately 25% of transcripts will be double coded to ensure the appropriateness of coding. A third coder will resolve any discrepancies. The criteria included in consolidated criteria for reporting qualitative research (COREQ), a 32 item checklist that will be used to conduct and report this study (Tong *et al.*, 2007).

### Component 4: empirical feasibility assessment study

#### Assessing empirical feasibility of evidence-based GPPAS interventions

This study would seek to appraise AMS strategies as part of the designed GPPAS model by stakeholders to identify feasible strategies for collaboration. Upon completion of this study, we would be able to prioritise AMS strategies and refine potential models for implementing AMS by increased collaboration between GPs and CPs.

## Methods

A list of AMS strategies for feasibility assessment will be identified and prioritised from the results of study components from 1 to 3. The list of suggested interventions will be discussed in collaboration between the NCAS and DGP, reviewed by the AMS researchers (DM, DCMK, and KT) and primary care researchers of DGP. All the feedback and suggestions for rephrasing the suggested interventions will be considered. Suggestions will be excluded if they are considered unfeasible.

An online survey will be used for the stakeholders to empirically assess the implementation feasibility of evidence-based AMS strategies using the APEASE criteria (Jolliffe, 2011): Affordability (is an intervention affordable?), Practicability (can it be delivered easily?), Effectiveness (is it likely to be effective?), Acceptability (is it acceptable to staff?), Side effects and safety (is it safe to implement?), and Equity (can it avoid inequalities in patient care?). Stakeholders from GP, pharmacy, academia, and primary health care network organisations (Royal Australian College of General Practitioners (RACGP), Primary Health Network (PHN), Pharmaceutical Society of Australia (PSA), Pharmacy Guild, and Department of Health) will be identified by the NCAS and DGP research team.

We will calculate the number of responses for each intervention, the maximum possible APEASE score for each intervention, and the actual APEASE score for each intervention. The percentage of the maximum possible APEASE score will be obtained for each intervention and will be calculated to allow comparison between interventions. Intervention will be scored based on the percentage of the maximum APEASE score to identify feasible interventions which have been rated highly.

## Discussion

**Figure 1.**
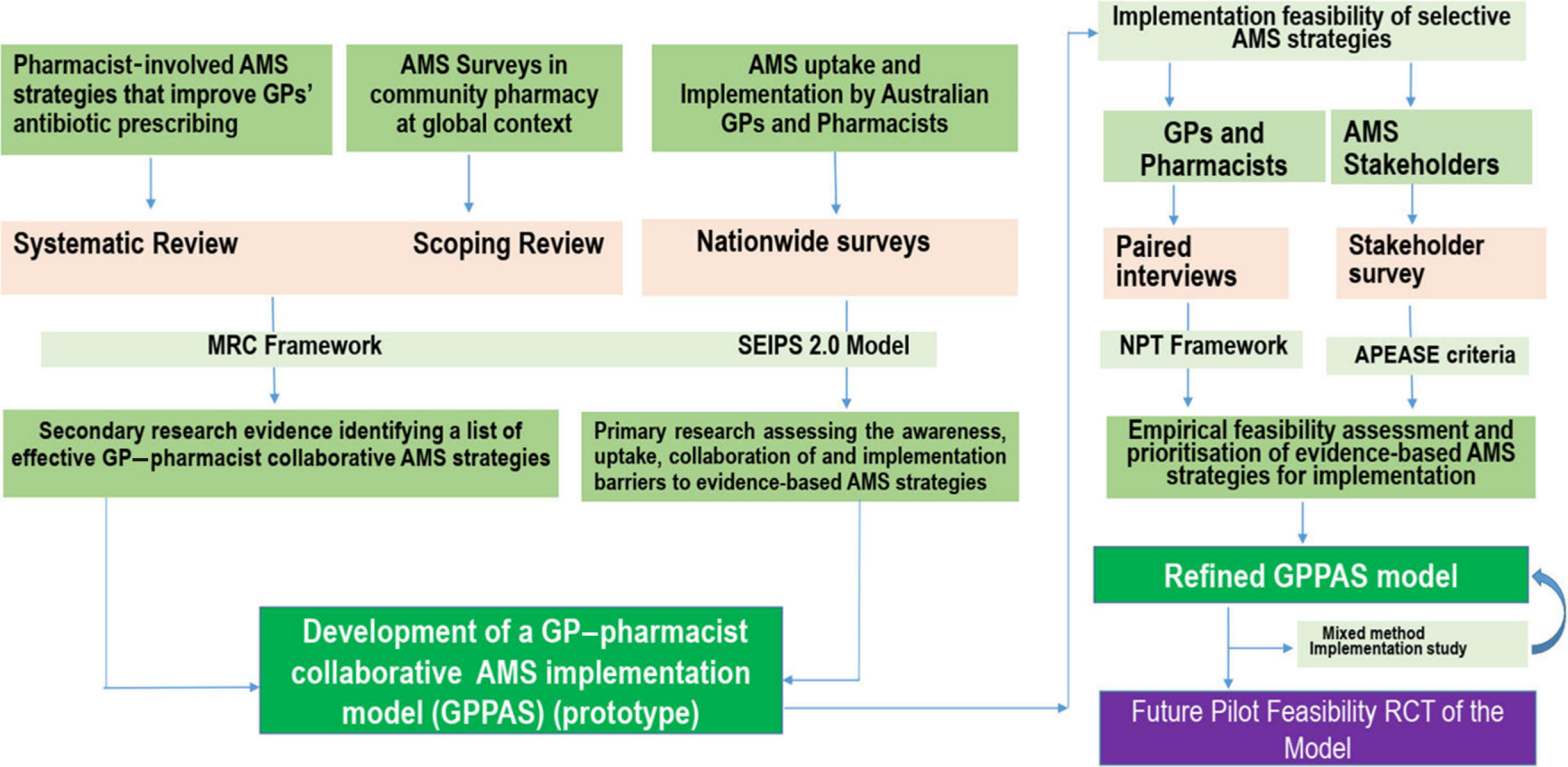
Process of development of GPPAS model.

In view of antimicrobial overprescribing and increasing AMR, the implementation of AMS programs in primary care is critically important. We develop an evidence-based and theory-informed GPPAS model to foster AMS implementation at the context of Australian primary care. Figure 1 shows the process of development of GPPAS model.

### Amalgamation of study components to design a GPPAS model and future evaluation

Our starting point was the evidence that certain the effectiveness of AMS interventions when implemented by GP–pharmacist collaboration. We will identify such effective AMS interventions that are not widely used in the context and also detect key barriers and facilitators to using these interventions. This will help us to create a logic model (in the form of a diagram) where we would put all the relevant determinants influencing the uptake of intervention in practice. Surveys, factor analyses, and interviews will chronically assist to identify the determinants. We will then systematically identify what we can realistically target to increase the uptake of the interventions, and address related implementation barriers. Thus, the GPPAS model will be consisting of intervention components, defined activities, and checklist for GPs and pharmacists, implementation needs and predicted outputs and impact. TIDieR (Template for Intervention Description and Replication) checklist will allow to better describe the interventions including the contents. SEIPS 2.0 model will help identify the GPPAS model components and the determinants that would influence the implementation of GPPAS model. The NPT will guide the theoretical underpinning to design the complex interventions of the GPPAS model. Furthermore, the APEASE criteria as defined in the Behavior Change Wheel (Affordability, Practicability, Effectiveness, and cost-effectiveness, Acceptability, Side-effects and safety, Equity) will be used to guide the selection of intervention options appropriate, its content, and implementation options. All authors will discuss to come into consensus about the draft of the GPPAS interventions and implementation strategies that would be practical and economic in primary care settings. This systematic process of development will involve multiple discussion sessions among multidisciplinary authors and AMS researchers of the National Centre for Antimicrobial Stewardship (NCAS).

For future steps, we may organize workshops including practitioners and stakeholders to get feedback on the designed GPPAS model for refining the model if required. In a separate step, we will then try out GPPAS interventions recruiting GP practices and community pharmacies to learn practical implementation and see how it works. We expect that we will be able to design the GPPAS model with our planned study but that might need further work for contextual adaptation and refinement. In this regard, we will continue our attempts to conduct mixed method implementation study to refine GPPAS model components and see its influence in the uptake of GPPAS interventions by GPs and CPs in future. Pragmatically, the designing and testing of the model would be an iterative process to finally establish the model and ensure team functioning to foster AMS in primary care. Given we have enough evidence, we will propose a feasibility randomized controlled trial (RCT) study to assess the effectiveness, feasibility, and acceptability of the GPPAS model in optimizing antimicrobial use and prevent AMR.

## Strength

This work describes the initial development phase of a GPPAS model to foster AMS implementation by collaboration between GPs and pharmacists, in ways that are effective and feasible in primary care settings. Our research identifies gaps in evidence of how GPs and CPs can work together to successfully implement AMS. AMS survey tools can be used as an important AMS resource in primary care to monitor and evaluate the uptake of AMS programs, AMS implementation status, and barriers over time. This study will also highlight the challenges of introducing AMS in Australian general practices and community pharmacies. In particular, the attitudinal exploration of GPs and CPs towards CP led interventions on the choice, dose, and dose regimen of antimicrobial prescriptions, collaborative AMS approaches, and relevant policy changes would be an important observation to explain the opportunities of collaboration in AMS in future. Additionally, the perception regarding the AMS system structure such as the use of ‘My Health Records’ (Hemsley *et al.*, 2018), eTG and telehealth will help to predict implementation feasibility of some evidence-based AMS strategies. Eventually, assimilation of the results of quantitative (practice and attitudinal issues), qualitative (barriers and facilitators), attitudinal modeling, and empirical feasibility assessment will be a strong scientific evidence to design a GPPAS model in the context of Australian primary care. Lastly, the design of GPPAS model is guided by the established theoretical frameworks, MRC, and NPT.

## Limitation

There are some limitations of this study. Firstly, from the perspective of logistics and costs issues, survey respondents will not be selected from the general practice or community pharmacy facilities where antimicrobial prescribing and dispensing are higher or prescription are mostly not adherent to Therapeutic Guideline (Antibiotic). These factors may have a confounding effect on the responses. Secondly, dissemination of AMS concept, implementation of AMS strategies, and AMS-related guidance and policies may vary from practice to practice, pharmacy to pharmacy and even state to state, therefore, responses may be confounded by the AMS implementation rate. The design of this model would not be based on the real life implementation study and RCT trial. However, our development would lead to design these trials in future.

## Conclusion

The purpose of this study is to advance the knowledge of AMS implementation by collaboration between GPs and CPs, and the delivery of antimicrobial care services for patients with infections in primary care. This study will identify the needs and an opportunity to foster AMS implementation with greater involvement of GPs and CPs. Eventually, the results will assist in designing a GPPAS model for future implementation and a pilot feasibility assessment in Australia. The GPPAS model that we develop, might have the potential to save the lives of antibiotics, safeguard patients and prevent AMR by securing a collaborative antimicrobial decision and care by GPs and CPs.

## Dissemination

Research outputs will be widely disseminated through peer reviewed journals, and local, national and international conferences and seminars. Our prior systematic review results had been orally presented to NAPCRG conference 2018 (Chicago, USA), Primary Health Care Research and Information Service (PHCRIS) conference 2018 (Melbourne, Australia), and NCAS primary care AMS seminar (Melbourne, Australia). Scoping review has been presented at the Society for Academic Primary Care (SAPC) conference 2019 (presentation session: 3B.3) in Exeter in the UK.

## Supplementary material

For supplementary material accompanying this paper visit https://doi.org/10.1017/S1463423620000687. click here to view supplementary material
