# Supplementary material for: Development of an antimicrobial stewardship implementation model involving collaboration between general practitioners and pharmacists: GPPAS study in Australian primary care
Source: Prim Health Care Res Dev. 2021 Jan 28;22:e2. doi: 10.1017/S1463423620000687 (PMC8057431; doi:10.1017/S1463423620000687)
Supplement: Supplementary file 1 [file S1463423620000687sup.zip › S1463423620000687sup003.docx]

**Additional file 3:** Recruitment and sampling of GPs and community pharmacists

| **A. GP- survey** | | | | | | | | | |
| --- | --- | --- | --- | --- | --- | --- | --- | --- | --- |
| Australia | 34, 606 GPs | | | | | | | | |
| Stratification by territories | NSW | QLD | VIC | ACT | SA | WA | TAS | NT | Total |
|  | 12153 | 7277 | 8939 | 555 | 2845 | 3805 | 997 | 2956 | 36937 |
| Simple random selection of GPs | 988 | 591 | 728 | 46 | 233 | 309 | 80 | 25 | 3000 |
| Sample percentage | 32.9 | 19.7 | 24.2 | 1.5 | 7.7 | 10.3 | 2.7 | 0.8 | 100% |
| Expected response rate from invited samples | 32.9 | 19.7 | 24.2 | 1.5 | 7.7 | 10.3 | 2.7 | 0.8 |  |
| **B. Pharmacy-survey** | | | | | | | | | |
| Australia | 5700 community pharmacies | | | | | | | | |
| Stratification by territories | NSW | QLD | VIC | ACT | SA | WA | TAS | NT | Total |
|  | 1630 | 1094 | 1436 | 97 | 667 | 393 | 302 | 74 | 5700 |
| Simple random selection of community pharmacies | 618 | 415 | 544 | 36 | 253 | 149 | 117 | 28 | 2160 |
| Sample percentage | 28.6 | 19.2 | 25.2 | 1.7 | 11.7 | 6.9 | 5.4 | 1.3 | 100% |
| Expected response rate from invited samples | 28.6 | 19.2 | 25.2 | 1.7 | 11.7 | 6.9 | 5.4 | 1.3 |  |
