# Supplementary material for: Development of an antimicrobial stewardship implementation model involving collaboration between general practitioners and pharmacists: GPPAS study in Australian primary care
Source: Prim Health Care Res Dev. 2021 Jan 28;22:e2. doi: 10.1017/S1463423620000687 (PMC8057431; doi:10.1017/S1463423620000687)
Supplement: Supplementary file 1 [file S1463423620000687sup.zip › S1463423620000687sup001.docx]

**Additional file 1:** GP-AMS survey instrument

| Please tick the option that best describes your opinion | | | | | | | | | | | | | |
| --- | --- | --- | --- | --- | --- | --- | --- | --- | --- | --- | --- | --- | --- |
| Section A  Demographic information | | | | | | | | | | | | | |
| 1 | Are you? | | Male | Female | | | | | | | | | |
| 2 | Your highest educational degree | | B.Med Science | MBBS | | MD | | | | Masters | | PhD | |
| 3 | No. of years in practice as a GP | |  ≤5 | 6-10 | | >10 | | | | | | | |
| 4 | Your current practice location | | Metro | Regional | | Rural | | | |  Remote | | | |
| 5 | Main state or territory where you work | | NSW QLD VIC ACT SA WA TAS  NT | | | | | | | | | | |
| 6 | Was your medical training completed outside of Australia? | | Yes | No | | If so, where- | | |  | | | | |
| 7 | Have you completed the National Prescribing Service (NPS) Medicine Wise antimicrobial prescribing courses? | | Yes | No | | I am not aware of these courses | | | | | | | |
| Section B  Perceived awareness of antimicrobial stewardship (AMS) | | | | | **Strongly**  **agree** | | **Agree** | **Neither agree nor disagree** | | | **Disagree** | | **Strongly**  **disagree** |
| 1 | | I am familiar with the term antimicrobial stewardship (AMS) | | |  | |  |  | | |  | |  |
| 2 | | AMS programs in my practice will significantly reduce inappropriate use of antimicrobials | | |  | |  |  | | |  | |  |
| 3 | | AMS programs will reduce health care costs associated with infections | | |  | |  |  | | |  | |  |
| 4 | | Individual efforts at AMS have minimal impact on the problem of antimicrobial resistance | | |  | |  |  | | |  | |  |
| 5 | | I require adequate training to undertake AMS in my practice | | |  | |  |  | | |  | |  |
| Section C  My current approach to prescribing of antimicrobials for my patients | | | | | **Always** | | **Often** | **Occasionally** | | | **Rarely** | | **Never** |
| 1 | | I use national antimicrobial guidelines when considering how to treat common infections | | |  | |  |  | | |  | |  |
| 2 | | I use back-up or delayed prescribing strategies for antimicrobial prescribing when appropriate | | |  | |  |  | | |  | |  |
| 3 | | I record the clinical indication for the antimicrobial(s) prescribed in the clinical records | | |  | |  |  | | |  | |  |
| 4 | | I educate my patients or their carers about unintended consequences of antimicrobial use like antimicrobial resistance, impact on gut microbiota etc. | | |  | |  |  | | |  | |  |
| 5 | | I use rapid point-of-care testing to guide clinical decision making whether to prescribe an antibiotic in treating a patient with infection like pharyngitis or the flu | | |  | |  |  | | |  | |  |
| 6 | | I share patient information leaflets about infections when I counsel my patients or carers who require antimicrobials or may have an infection | | |  | |  |  | | |  | |  |
| 7 | | I am being involved in a practice that undertakes audit on antimicrobial prescribing and provide feedback outcome of the audit | | |  | |  |  | | |  | |  |
| 8 | | I review and discuss antimicrobial prescribing at my practice in comparison to peer prescribers at least once a year | | |  | |  |  | | |  | |  |
|  | | | | | | | | | | | | | |
|  | | **Section D**  **Strengthening GP-pharmacist collaborations in AMS** | | | **Strongly**  **agree** | | **Agree** | **Neither agree**  **nor disagree** | | | **Disagree** | | **Strongly**  **disagree** |
| 1 | | Improving AMS in the community will need a policy that supports better collaboration between general practice and pharmacy | | |  | |  |  | | |  | |  |
| 2 | | GPs should be receptive to pharmacists providing advice about the choice of antimicrobial prescribed | | |  | |  |  | | |  | |  |
| 3 | | GPs should be receptive to pharmacists making recommendations in consultation to the doses or formulations of the antimicrobial prescribed | | |  | |  |  | | |  | |  |
| 4 | | An electronic prescription exchange technology between GP and pharmacy should be introduced for reviewing the appropriateness of antimicrobial prescriptions | | |  | |  |  | | |  | |  |
| 5 | | Pharmacists with knowledge of antimicrobials and infections should attend regular group meetings of GPs within general practice clinic to discuss antimicrobial pharmacotherapy | | |  | |  |  | | |  | |  |
| 6 | | A pharmacist co-located within general practice can help optimise antimicrobial therapy of patients with infections | | |  | |  |  | | |  | |  |
| 7 | | The “My Health Record” could improve communication between GPs and community pharmacists about antimicrobial prescriptions | | |  | |  |  | | |  | |  |
| Section E  Building stronger AMS intervention in the general community | | | | | **Strongly**  **Agree** | | **Agree** | **Neither agree**  **nor disagree** | | | **Disagree** | | **Strongly**  **disagree** |
| 1 | | I would be willing to participate in a program of training focused on AMS | | |  | |  |  | | |  | |  |
| 2 | | I support the introduction of standard guidelines for GPs to assist in the implementation of AMS programs | | |  | |  |  | | |  | |  |
| 3 | | I support a policy that limits the prescribing of selected antimicrobial to certain clinical conditions | | |  | |  |  | | |  | |  |
| 4 | | I support a policy that supports mandatory documentation of the clinical indication (in the notes and on the script) for antimicrobial prescribing | | |  | |  |  | | |  | |  |
| 5 | | I support the involvement of a specialist physician and a pharmacist in providing individualised antimicrobial prescribing advice and feedback to GPs | | |  | |  |  | | |  | |  |
| 6 | | Professional organisations (e.g. RACGP) should define my roles and responsibilities regarding AMS in my practice | | |  | |  |  | | |  | |  |
| 7 | | I support a system that better supports the integration of eTG (Therapeutic Guidelines) with prescribing software | | |  | |  |  | | |  | |  |

What do you believe are the major barriers and facilitators to implementing AMS in your practice?

**Barriers**………………………………………………………………………………………………………………………………………………………………………………………………………………………………………………………………………………………………………………………………………………………………………………………………………………………………………………………………………………………………………………………………………………………………………………………………………………………………………………………………………………………………………………………………………….........................................................................................................................................................................................................................................................................................................................................................................................................................................................................................................................................................................................................................................................................................................................................................................................................................................................................................................................................................................................................................................................................................................................................................................................................................................................................................................................................................................................................................................................................................................................................................................................................................................................................................................................................................

**Facilitators:** ……………………………………………………………………………………………………………………………………………………………………………………………………………………………………………………………………………………………………………………………………………………….…………………………………………………………………………………………………………………………………………………………………………………….…………………………………………………………………………………………………………………………………………………………………………………….…………………………………………………………………………………………………………………………………………………………………………………….…………………………………………………………………………………………………………………………………………………………………………………….…………………………………………………………………………………………………………………………………………………………………………………….…………………………………………………………………………………………………………………………………………………………………………………….

**I would be interested in participating in a brief interview of approximately 30 minutes on this topic:**

**☐ Yes –My contact details are: Name:**

**Address:**

**Email:**

**Best contact No:**

**☐ No**
