# Supplementary material for: Development of an antimicrobial stewardship implementation model involving collaboration between general practitioners and pharmacists: GPPAS study in Australian primary care
Source: Prim Health Care Res Dev. 2021 Jan 28;22:e2. doi: 10.1017/S1463423620000687 (PMC8057431; doi:10.1017/S1463423620000687)
Supplement: Supplementary file 1 [file S1463423620000687sup.zip › S1463423620000687sup002.docx]

| Please tick the option that best describes your opinion | | | | | |
| --- | --- | --- | --- | --- | --- |
| Section A  Demographic information | | | | | |
| 1 | Are you? | Male | Female |  | |
| 2 | Your highest educational degree in pharmacy | Bachelor | Masters | PhD | Other |
| 3 | Your current practice location | Metro | Regional | Rural | Remote |
| 4 | Main state or territory where you work | NSW QLD VIC ACT SA WA TAS  NT | | | |
| 5 | No. of years in practice as a community pharmacist | ≤5 | 6-10 | >10 |  |
| 6 | Do you work in a sector other than community pharmacy? | Yes No | | | |
| 7 | Where applicable, please indicate and provide the % of your working time in each sector below  Community  _____% Hospital  _____% HMR/RMMR  _____% General Practice  _____%  Industry  _____% Academia  _____% Others:  Please specify _______ | | | | |
| 8 | Was your pharmacy training completed outside of Australia? | Yes | No | If so, where- | |
| 9 | Have you completed the National Prescribing Service (NPS) Medicine Wise antimicrobial modules to improve antimicrobial use in patients? | Yes | No | I am not aware of these modules | |

| Section B  Perceived awareness of antimicrobial stewardship (AMS) | | Strongly  agree | Agree | Neither agree nor disagree | Disagree | Strongly  disagree |
| --- | --- | --- | --- | --- | --- | --- |
| 1 | I am familiar with the term antimicrobial stewardship (AMS) |  |  |  |  |  |
| 2 | AMS programs in my practice will significantly reduce inappropriate use of antimicrobials |  |  |  |  |  |
| 3 | AMS programs will reduce health care costs associated with infections |  |  |  |  |  |
| 4 | Individual efforts at AMS have minimal impact on the problem of antimicrobial resistance |  |  |  |  |  |
| 5 | I require adequate training to undertake AMS in my pharmacy |  |  |  |  |  |
| Section C  My current approach to dispensing of antimicrobials for my patients | | **Always** | **Often** | **Occasionally** | **Rarely** | **Never** |
| 1 | I use national antimicrobial guidelines when dispensing antimicrobials to my patients |  |  |  |  |  |
| 2 | I communicate with my patient’s or carer’s general practitioner (GP) if I have a reason to believe that the choice of antimicrobial may not be optimal in terms of the patient’s infection |  |  |  |  |  |
| 3 | I consider clinical safety parameters like drug interactions, adverse drug reactions, and allergies before dispensing a prescribed antimicrobial |  |  |  |  |  |
| 4 | I educate my patients or their carers about unintended consequences of antimicrobial use like antimicrobial resistance, impact on gut microbiota etc. |  |  |  |  |  |
| 5 | Before dispensing antimicrobial repeat prescription for ‘the flu’ or an upper respiratory tract infection, I recommend my patient or carer to consult a GP |  |  |  |  |  |
| 6 | I use rapid point-of-care testing to guide my clinical decision about whether to dispense an antibiotic to the patients with pharyngitis or the flu |  |  |  |  |  |
| 7 | I share patient information leaflets about infections when I counsel my patients or carers who require antimicrobials or may have an infection |  |  |  |  |  |
| 8 | I educate my patients or carers on how to prevent vaccine-preventable infections (e.g. influenza) |  |  |  |  |  |
| 9 | I counsel my patients or their carers, on how to take their antimicrobial(s) including dose and frequency, the possible side effects, and the importance of completing the course of antimicrobial(s) prescribed |  |  |  |  |  |
| 10 | I check whether antimicrobial prescription is compliant with therapeutic guideline before dispensing antimicrobials |  |  |  |  |  |
| 11 | I feel confident to check guideline compliance of antimicrobial prescriptions |  |  |  |  |  |
|  | **Section D**  **Strengthening GP-pharmacist collaborations in AMS** | **Strongly**  **agree** | **Agree** | **Neither agree**  **nor disagree** | **Disagree** | **Strongly**  **disagree** |
| 1 | Improving AMS in the community will need a policy that supports better collaboration between general practice and pharmacy |  |  |  |  |  |
| 2 | GPs should be receptive to pharmacists providing advice about the choice of antimicrobial prescribed |  |  |  |  |  |
| 3 | GPs should be receptive to pharmacists making recommendations in consultation to the doses or formulations of the antimicrobial prescribed |  |  |  |  |  |
| 4 | An electronic prescription exchange technology between GP and pharmacy should be introduced for reviewing the appropriateness of antimicrobial prescriptions |  |  |  |  |  |
| 5 | Pharmacists with knowledge of antimicrobials and infections should attend regular group meetings of GPs within general practice clinic to discuss antimicrobial pharmacotherapy |  |  |  |  |  |
| 6 | A pharmacist co-located within general practice can help optimise antimicrobial therapy of patients with infections |  |  |  |  |  |
| 7 | The “My Health Record” could improve communication between GPs and community pharmacists about antimicrobial prescriptions |  |  |  |  |  |
| Section E  Building stronger AMS intervention in the general community | | **Strongly**  **Agree** | **Agree** | **Neither agree**  **nor disagree** | **Disagree** | **Strongly**  **disagree** |
| 1 | I would be willing to participate in a program of training focused on AMS |  |  |  |  |  |
| 2 | I support the introduction of standard guidelines for community pharmacists to assist in the implementation of AMS programs |  |  |  |  |  |
| 3 | I support a policy that requires community pharmacists keeping updated on current practice guidelines for antimicrobial use |  |  |  |  |  |
| 4 | I support a policy that limits accessibility of some antimicrobials in the community |  |  |  |  |  |
| 5 | I support the involvement of a specialist physician and a pharmacist providing individualised antimicrobial prescribing advice and feedback to GPs |  |  |  |  |  |
| 6 | Professional pharmacy organisations should define my roles and responsibilities regarding AMS activities |  |  |  |  |  |
| 7 | Patients leaflets on antimicrobial awareness should be introduced into my pharmacy |  |  |  |  |  |
| 8 | I support a policy that limits the dispensing of repeat antimicrobial prescriptions |  |  |  |  |  |

What do you believe the major barriers and facilitators to implementing AMS in your pharmacy?

**Barriers**:…………………………………………………………………………………………………………………………………………………………………………………………………………………………………………………………………………………………………………………………………………………………………………………………………………………………………………………………………………………………………………………………………………………………………………………………………………………………………………………………………………………………………………………………………………………………………………………………………………………………………………………………………………………………………………………………………………………………………………………………………………………………………………………………………………………………………………………………………………**Facilitators**………………………………………………………………………………………………………………………………………………………………..……………………………………………………………………………………………………………………………………………………………………………………………………………………………………………………………………………………………………………………………………………………………………………………………………………………………………………………………………………………………………………………………………

I would be interested in participating in a brief interview of approximately 30 minutes on this topic-

☐ Yes –My contact details are: Name: Address:

☐ No Email: Best contact No:

Please enter your email address if you would like to go into the draw to win 1 of 4 $100 gift vouchers ………………………..
